# Clinically relevant reductions in HbA_1c_ without hypoglycaemia: results across four studies of saxagliptin

**DOI:** 10.1111/ijcp.12212

**Published:** 2013-06-24

**Authors:** C S Karyekar, R Frederich, S Ravichandran

**Affiliations:** Bristol-Myers SquibbPrinceton, NJ, USA

## Abstract

**Background**In four 24-week controlled studies, the antihyperglycaemic efficacy of saxagliptin was demonstrated in patients with type 2 diabetes mellitus as add-on therapy to glyburide, a thiazolidinedione, or metformin, and when used in initial combination with metformin vs. metformin monotherapy in drug-naive patients.

**Methods**Data from these studies were analysed to compare the proportions of patients who achieved specific reductions from baseline in glycated haemoglobin [HbA_1c_; reductions of ≥ 0.5% and ≥ 0.7% in all studies (prespecified); reductions ≥ 1.0% in the add-on studies and ≥ 1.0% to ≥ 2.5% in the initial combination study (*post hoc*)] for saxagliptin vs. comparator at week 24. We report overall rates of glycaemic response defined by these reductions in HbA_1c_ and rates of response without experiencing hypoglycaemia.

**Results**Large glycaemic response rates were higher with saxagliptin 2.5 and 5 mg/day than with comparator (HbA_1c_ ≥ 1.0%, 31.7–50.3% vs. 10.3–20.0%) as add-on therapy and higher with saxagliptin 5 mg/day as initial combination with metformin than with metformin monotherapy (HbA_1c_ ≥ 2.0%, 68.3% vs. 49.8%) in drug-naive patients. Addition of saxagliptin was associated with a low incidence of hypoglycaemia; overall response rates and response rates excluding patients who experienced hypoglycaemia were similar. Analysis of several demographic and baseline clinical variables revealed no consistent correlations with response to saxagliptin.

**Conclusions**Whether receiving saxagliptin as an add-on therapy to glyburide, a thiazolidinedione, or metformin or in initial combination with metformin, a greater percentage of patients achieve clinically relevant large reductions in HbA_1c_ vs. comparator, with a low incidence of hypoglycaemia.

## What's known

- The risk of developing complications of diabetes increases with increasing levels of glycaemia.
- Greater reductions in hyperglycaemia lower the risk of complications.

## What's new

- The proportions of patients who achieved large reductions in HbA_1c_ (responders) were significantly higher with saxagliptin as an add-on to another antihyperglycaemic drug or in initial combination with metformin than with the comparator regimens.
- Hypoglycaemia incidence is low with saxagliptin; responder rates excluding patients who experience hypoglycaemia were also significantly higher with saxagliptin.
- Response to saxagliptin did not consistently correlate with any demographic or baseline characteristic analysed.

## Introduction

Glycaemic control to a specific recommended target is an important goal in the management of type 2 diabetes mellitus (T2DM) to minimise the risk of microvascular and macrovascular complications 1–5. However, patients do not always reach the target glycated haemoglobin (HbA_1c_) levels (< 6.5% or < 7.0%) recommended for most patients by the American Association of Clinical Endocrinologists (AACE) 6 and the American Diabetes Association (ADA)/European Society for the Study of Diabetes (EASD) 7 with antihyperglycaemic therapy. To reduce HbA_1c_ in T2DM, a variety of antihyperglycaemic drugs are available, utilising different mechanisms of action and offering different degrees of effectiveness and safety. Metformin is the most commonly used first-line agent and works primarily by inhibiting hepatic gluconeogenesis and enhancing skeletal muscle uptake of glucose 8–9. Insulin secretagogues, including sulphonylureas, are effective but may cause hypoglycaemia and weight gain via excessive stimulation of insulin and tend to become less effective over time 10. Thiazolidinediones (TZDs) improve glycaemic control by increasing insulin sensitivity but are associated with weight gain and oedema and increased risk of congestive heart failure 7–11. Dipeptidyl peptidase-4 (DPP-4) inhibitors are newer agents that act by stimulating glucose-dependent insulin secretion 12–13. They offer a low risk of hypoglycaemia, are not associated with weight gain 14, and fulfil the need for safe and effective antihyperglycaemic drugs.

Irrespective of the choice of therapy, reductions in HbA_1c_ lower the risk of complications. For example, a report from the United Kingdom Prospective Diabetes Study (UKPDS) showed that each 1% decrease in HbA_1c_ was associated with significant reductions of 21% in diabetes-related deaths, 14% in myocardial infarction and 37% in microvascular complications 4. Thus, it is valuable to understand the magnitude of HbA_1c_ reductions that might occur with antihyperglycaemic therapy, even in patients for whom reaching target HbA_1c_ levels is challenging. Furthermore, an understanding of which patient groups may experience the greatest reduction in HbA_1c_ levels may help guide physicians in choosing optimal treatment for patients with T2DM.

Dipeptidyl peptidase-4 inhibitors are known to provide mean reductions from baseline in HbA_1c_ in the range of 0.5–0.8% when used as monotherapy in conjunction with diet and exercise or as combination therapy with metformin, a sulphonylurea, or a TZD 15–23. However, a subset of patients can achieve larger reductions in HbA_1c_. In addition, the favourable safety and tolerability profile of these agents and their oral route of administration may facilitate adherence to treatment 24. This would be anticipated to improve ‘real world’ glycaemic control. For these reasons, the AACE and ADA/EASD treatment recommendations consider DPP-4 inhibitors as one of the first-line therapies in patients in whom metformin is contraindicated, and one of the first choices in combination with metformin 7–25.

The DPP-4 inhibitor, saxagliptin, is indicated as an adjunct to diet and exercise to improve glycaemic control in adults with T2DM 26. Randomised phase 3 trials have shown that reductions in HbA_1c_ were significantly greater with saxagliptin 5 mg once daily vs. placebo or comparator as an add-on to metformin 16, the sulphonylurea glyburide 15, or a TZD (pioglitazone or rosiglitazone) 17; and significantly greater with saxagliptin 5 mg as initial combination with metformin vs. metformin monotherapy in drug-naive patients 18. Except for the study of add-on to the sulphonylurea, saxagliptin was not associated with an increased risk of hypoglycaemia.

In these phase 3 trials of saxagliptin, reductions from baseline in HbA_1c_ of 0.5% and 0.7% were assessed as prespecified end-points. Understanding the potential of saxagliptin to achieve large decreases in HbA_1c_ could be more meaningful, especially in patients who rarely achieve glycaemic targets. Therefore, a *post hoc* analysis of data from these trials to evaluate the ability of saxagliptin to produce clinically relevant and larger-than-prespecified mean reductions in HbA_1c_ (≥ 1.0% in the add-on studies; ≥ 1.0% to ≥ 2.5% in the initial combination study) was conducted. As large reductions in HbA_1c_ could potentially be associated with a higher risk of hypoglycaemia, we defined and evaluated antihyperglycaemic effects as successful when defined reductions in HbA_1c_ were achieved without incurring hypoglycaemia. Furthermore, demographic and baseline clinical variables were examined to identify possible predictors of large glycaemic response to treatment with saxagliptin.

## Methods

The methods for each of these studies have been previously published 15–18. Participants were adults aged 18–77 years with a diagnosis of T2DM and baseline HbA_1c_ indicating inadequate glycaemic control within defined limits: 7–10% in the study of saxagliptin add-on to metformin 16, 8–12% in the study of initial combination with metformin 18, 7.5–10% in the study of saxagliptin add-on to glyburide 15 and 7–10.5% in the study of saxagliptin add-on to TZD 17. Other study entry criteria included body mass index (BMI) < 40 kg/m^2^ (extended to < 45 kg/m^2^ in the study of add-on to a TZD) and no contraindications to therapy with a DPP-4 inhibitor.

In the add-on to metformin study 16, patients receiving stable total daily doses of metformin 1500–2550 mg/day at study entry were randomly assigned to receive saxagliptin 2.5, 5, or 10 mg once daily or placebo. In the add-on studies with glyburide 15 or a TZD 17, patients were randomly assigned to receive saxagliptin 2.5 or 5 mg once daily or placebo along with continued use of the background agent (in the placebo arms, dosing was uptitrated for glyburide but kept at the regimen in use at baseline for the TZD). In the study of initial combination with metformin 18, drug-naive patients were randomly assigned to receive metformin, titrated from 500 mg/day to a target dosage of 2000 mg/day, as monotherapy or in combination with saxagliptin 5 or 10 mg once daily; patients in a fourth treatment arm received saxagliptin 10 mg once daily as monotherapy.

### Analysis

For each of the studies in this analysis, the primary end-point was adjusted mean change from baseline to week 24 in HbA_1c_. Secondary end-points generally included adjusted mean change from baseline to week 24 in fasting plasma glucose, adjusted mean change from baseline to week 24 in postprandial glucose, and the proportion of subjects achieving HbA_1c_ < 7%. Glycaemic response levels were also defined by specific reductions in HbA_1c_ after 24 weeks of treatment. In the original studies, percentages of patients achieving reductions in HbA_1c_ of ≥ 0.5% and ≥ 0.7% were prespecified as additional end-points.

The present *post hoc* analyses include data only from patients who received the 2.5- and 5-mg doses of saxagliptin, which are the doses approved by the United States Food and Drug Administration and the European Union. The proportions of patients in each treatment group who achieved larger reductions in HbA_1c_ were evaluated in each study. For the add-on studies, reductions in HbA_1c_ of ≥ 1.0% were assessed; for the study of initial combination therapy with metformin in drug-naive patients, HbA_1c_ reductions of ≥ 1.0%, ≥ 1.5%, ≥ 2.0% and ≥ 2.5% were assessed. More stringent criteria were used in the initial combination study because large reductions in HbA_1c_ would be expected.

In addition to reporting overall rates of response defined by these specific reductions in HbA_1c_, we report response rates for patients who did not experience hypoglycaemia during treatment, counting patients who experienced one or more episodes of hypoglycaemia as non-responders. In these analyses, hypoglycaemia was based on reports of symptoms (with or without fingerstick confirmation).

For statistical analysis, last observed data were carried forward for participants who did not complete 24 weeks of treatment. Comparisons of the proportions of patients who achieved defined glycaemic response with saxagliptin vs. comparator were performed using Fisher's exact test and 95% exact CIs for the difference in proportions were calculated.

To explore correlations between baseline characteristics and decreases in HbA_1c_, either overall or for a specific treatment group, logistic regression was performed, using terms for treatment group, baseline characteristic and treatment group by baseline characteristic. For each of the four studies, odds ratios for the association of achieving a specific percentage decrease in HbA_1c_ for saxagliptin vs. comparator were calculated for 10 baseline covariates [sex, age, duration of diabetes, BMI and HbA_1c_ as categories; and quartiles for C-peptide area under the curve (AUC), insulin AUC, glucagon AUC, glucose AUC, and insulin secretion assessed as homeostasis model assessment 2 β-cell function (HOMA2-%β)], to identify characteristics that could correlate with glycaemic response (defined for this purpose as HbA_1c_ reduction ≥ 1% in the add-on studies and ≥ 2% in the initial combination study). Nominal p-values for the association of each baseline characteristic and baseline characteristic by treatment group with response rate were generated from the logistic analysis.

## Results

A total of 3382 patients participated in the four clinical studies. Baseline characteristics of the study populations have been previously published 15–18. Across the four studies, mean age of participants ranged from 52 to 55 years, mean duration of diabetes ranged from 1.4 to 7.1 years and mean BMI ranged from 29 to 32 kg/m^2^; men and women participated in roughly equal numbers, and most of the participants were white.

### Glycaemic response

**Figure 1:**
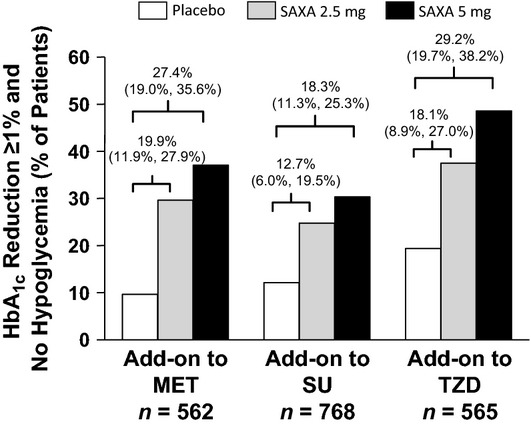
Large glycaemic response (HbA_1c_ reduction from baseline ≥ 1.0%)* in studies of saxagliptin as an add-on to another oral antihyperglycaemic drug. MET, metformin; SAXA 2.5, saxagliptin 2.5 mg once daily; SAXA 5, saxagliptin 5 mg once daily; SU, sulphonylurea (glyburide); TZD, thiazolidinedione (pioglitazone or rosiglitazone). *n*, overall number of patients randomised and treated. *Difference (95% CI) from placebo shown above bars

In the three add-on studies, in which saxagliptin was given at 2.5 and 5 mg once daily 15,16, glycaemic response rates were numerically higher with the 5-mg than with the 2.5-mg dose. In this *post hoc* analysis, the proportions of patients achieving predetermined glycaemic responses without incurring hypoglycaemia were higher with saxagliptin 2.5 and 5 mg once daily than with comparators in each of the individual studies (Tables 3). In the saxagliptin add-on to metformin study (Table 1, Figure 1), the rates of response defined by HbA_1c_ reduction ≥ 1% were 33.3% with saxagliptin 2.5 mg plus metformin and 39.8% with saxagliptin 5 mg plus metformin vs. 10.3% with placebo plus metformin [difference from placebo (95% CI) 23.0% (14.8%, 31.2%) and 29.5% (20.9%, 37.8%) for 2.5 and 5 mg, respectively]. When patients who experienced one or more hypoglycaemia events were removed from the analysis (5.2–7.8% in the saxagliptin plus metformin groups vs. 5.0% in the placebo plus metformin group), the rates of response were 29.6% for 2.5 mg and 37.l% for 5 mg vs. 9.7% for placebo [difference from placebo 19.9% (11.9%, 27.9%) and 27.4% (19.0%, 35.6%)].

**Table:** Glycaemic responses at 24 weeks with saxagliptin vs. placebo as an add-on to metformin*

		Response rates among all assessed patients		Response rates in patients who did not experience hypoglycaemia	
Criterion for response	Treatment groups	*n/N* (%)	Difference (95% CI) vs. PBO + MET	*n*/*N* (%)	Difference (95% CI) vs. PBO + MET
HbA_1c_ reduction from BL ≥ 1.0%	PBO + MET (*n *= 179)	18/175 (10.3)	–	17/175 (9.7)	–
	SAXA 2.5 + MET (*n *= 192)	62/186 (33.3)	23.0% (14.8%, 31.2%)	55/186 (29.6)	19.9% (11.9%, 27.9%)
	SAXA 5 + MET (*n *= 191)	74/186 (39.8)	29.5% (20.9%, 37.8%)	69/186 (37.1)	27.4% (19.0%, 35.6%)
HbA_1c_ reduction from BL ≥ 0.7%	PBO + MET (*n *= 179)	38/175 (21.7)	–	35/175 (20.0)	–
	SAXA 2.5 + MET (*n *= 192)	85/186 (45.7)	24.0% (14.3%, 33.3%)	75/186 (40.3)	20.3% (10.9%, 29.4%)
	SAXA 5 + MET (*n *= 191)	102/187 (51.1)	32.8% (23.1%, 42.0%)	95/186 (51.1)	31.1% (21.4%, 40.2%)
HbA_1c_ reduction from BL ≥ 0.5%	PBO + MET (*n *= 179)	47/175 (26.9)	–	43/175 (24.6)	–
	SAXA 2.5 + MET (*n *= 192)	105/186 (56.5)	29.6% (19.6%, 39.1%)	94/186 (50.5)	26.0% (16.0%, 35.4%)
	SAXA 5 + MET (*n *= 191)	118/187 (63.1)	36.2% (26.3%, 45.4%)	111/186 (59.7)	35.1% (25.2%, 44.3%)

BL, baseline; CI, confidence interval; HbA_1c_, glycated haemoglobin; MET, metformin; PBO, placebo; SAXA 2.5, SAXA 5, saxagliptin 2.5 or 5 mg once daily. *n*/*N*, number of responders/number assessed.

* Metformin continued at stable dosages of 1500–2550 mg/day.

**Table:** Glycaemic responses at 24 weeks with saxagliptin vs. placebo as an add-on to a sulphonylurea*

		Response rates among all assessed patients		Response rates in patients who did not experience hypoglycaemia	
Criterion for response	Treatment groups	*n*/*N* (%)	Difference (95% CI) vs. PBO + SU	*n*/*N* (%)	Difference (95% CI) vs. PBO + SU
HbA_1c_ reduction om BL ≥ 1.0%	PBO + SU (*n *= 267)	36/264 (13.6)	–	32/264 (12.1)	–
	SAXA 2.5 + SU (*n *= 248)	78/246 (31.7)	18.1% (10.9%, 25.2%)	61/246 (24.8)	12.7% (6.0%, 19.5%)
	SAXA 5 + SU (*n *= 253)	91/250 (36.4)	22.8% (15.4%, 30.0%)	76/250 (30.4)	18.3% (11.3%, 25.3%)
HbA_1c_ reduction from BL ≥ 0.7%	PBO + SU (*n *= 267)	57/264 (21.6)	–	47/264 (17.8)	–
	SAXA 2.5 + SU (*n *= 248)	105/246 (42.7)	21.1% (13.0%, 29.0%)	82/246 (33.3)	15.5% (8.0%, 23.1%)
	SAXA 5 + SU (*n *= 253)	123/250 (49.2)	27.6% (19.5%, 35.4%)	103/250 (41.2)	23.4% (15.6%, 31.0%)
HbA_1c_ reduction from BL ≥ 0.5%	PBO + SU (*n *= 267)	78/264 (29.5)	–	65/264 (24.6)	–
	SAXA 2.5 + SU (*n *= 248)	127/246 (51.6)	22.1% (13.6%, 30.3%)s	102/246 (41.5)	16.8% (8.6%, 24.8%)
	SAXA 5 + SU (*n *= 253)	145/250 (56.8)	27.3% (18.8%, 35.3%)	119/250 (47.6)	23.0% (14.7%, 31.0%)

BL, baseline; CI, confidence interval; HbA_1c_, glycated haemoglobin; PBO, placebo; SAXA 2.5, SAXA 5, saxagliptin 2.5 or 5 mg once daily; SU, sulphonylurea. *n*/*N *= number of responders/number assessed.

* Glyburide at the following dosages: 7.5 mg/day open-label; increased to 10 mg/day at randomisation in the placebo group by addition of 2.5 mg/day as part of blinded regimen; in all groups, adjustable down to 5 mg/day in case of hypoglycaemia or up to 15 mg/day at investigator's discretion.

**Table:** Glycaemic responses at 24 weeks with saxagliptin vs. placebo as an add-on to a thiazolidinedione*

		Response rates among all assessed patients		Response rates in patients who did not experience hypoglycaemia	
Criterion for response	Treatment groups	*n*/*N* (%)	Difference (95% CI) vs. PBO + TZD	*n*/*N* (%)	Difference (95% CI) vs. PBO + TZD
HbA_1c_ reduction from BL ≥ 1.0%	PBO + TZD (*n *= 184)	36/180 (20.0)	–	35/180 (19.4)	–
	SAXA 2.5 + TZD (*n *= 195)	76/192 (39.6)	19.6% (10.4%, 28.6%)	72/192 (37.5)	18.1% (8.9%, 27.0%)
	SAXA 5 + TZD (*n *= 186)	92/183 (50.3)	30.3% (20.7%, 39.3%)	89/183 (48.6)	29.2% (19.7%, 38.2%)
HbA_1c_ reduction from BL ≥ 0.7%	PBO + TZD (*n *= 184)	60/180 (33.3)	–	59/180 (32.8)	–
	SAXA 2.5 + TZD (*n *= 195)	102/192 (53.1)	19.8% (9.7%, 29.5%)	97/192 (50.5)	17.7% (7.7%, 27.4%)
	SAXA 5 + TZD (*n *= 186)	116/183 (63.4)	30.1% (19.9%, 39.7%)	112/183 (61.2)	28.4% (18.2%, 38.1%)
HbA_1c_ reduction from BL ≥ 0.5%	PBO + TZD (*n *= 184)	80/180 (44.4)	–	78/180 (43.3)	–
	SAXA 2.5 + TZD (*n *= 195)	117/192 (60.9)	16.5% (6.3%, 26.4%)	112/192 (58.3)	15.0% (4.8%, 24.9%)
	SAXA 5 + TZD (*n *= 186)	131/183 (71.6)	27.1% (17.1%, 36.6%)	127/183 (69.4)	26.1% (15.9%, 35.7%)

BL, baseline; CI, confidence interval; HbA_1c_, glycated haemoglobin; PBO, placebo; SAXA 2.5, SAXA 5, saxagliptin 2.5 or 5 mg once daily; TZD, thiazolidinedione.

* Pioglitazone continued at 4 or 8 mg/day or rosiglitazone continued at 30 or 45 mg/day.

In the saxagliptin add-on to the sulphonylurea glyburide study (Table 2, Figure 1), the rates of response defined by HbA_1c_ reduction ≥ 1% were 31.7% with saxagliptin 2.5 mg plus glyburide and 36.4% with saxagliptin 5 mg plus glyburide vs. 13.6% with placebo plus uptitrated glyburide [difference from placebo 18.1% (10.9%, 25.2%) and 22.8% (15.4%, 30.0%) for 2.5 and 5 mg, respectively]. Not counting patients who experienced one or more episodes of hypoglycaemia (13.3–14.6% in the saxagliptin plus glyburide groups vs. 10.1% in the placebo plus glyburide group), the rates of response were 24.8% and 30.4% vs. 12.1% [difference from placebo 12.7% (6.0%, 19.5%) and 18.3% (11.3%, 25.3%) for 2.5 and 5 mg, respectively].

In the saxagliptin add-on to a TZD study (Table 3, Figure 1), the rates of response defined by HbA_1c_ reduction ≥ 1% were 39.6% with saxagliptin 2.5 mg plus TZD and 50.3% with saxagliptin 5 mg plus TZD vs. 20.0% with placebo plus TZD [difference from placebo 19.6% (10.4%, 28.6%) and 30.3% (20.7%, 39.3%) for 2.5 mg and 5 mg, respectively]. Without patients who experienced one or more episodes of hypoglycaemia (2.7–4.1% in the saxagliptin plus TZD groups vs. 3.8% in the placebo plus TZD group), the rates of response were 37.5% and 48.6% vs. 19.4% [difference from placebo 18.1% (8.9%, 27.0%) and 29.2% (19.7%, 38.2%) for 2.5 mg and 5 mg, respectively].

**Figure 2:**
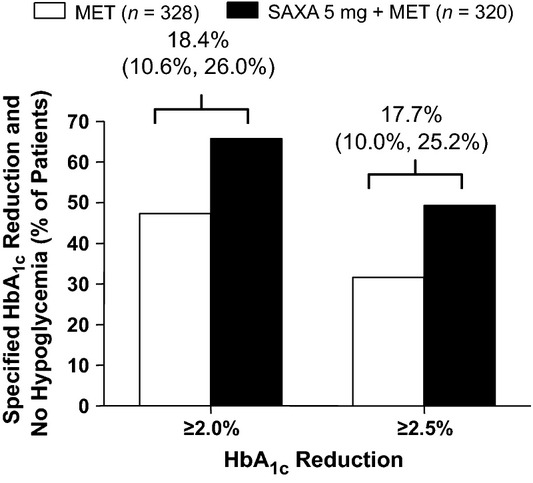
Large glycaemic response (HbA_1c_ reductions from baseline ≥ 2.0% and ≥ 2.5%)* in study of saxagliptin + metformin as initial combination therapy vs. metformin monotherapy in drug-naive patients. MET, metformin; SAXA 5,  saxagliptin 5 mg once daily. *n*, number of patients randomised and treated. *Difference (95% CI) from metformin monotherapy shown above bars

In the analysis of initial combination therapy with saxagliptin 5 mg and metformin in drug-naive patients (Table 4, Figure 2), rates of response defined by HbA_1c_ reduction ≥ 2.0% were 68.3% with saxagliptin plus metformin vs. 49.8% with metformin monotherapy [difference from metformin 18.5% (10.7%, 26.0%)]; rates of response defined by HbA_1c_ reduction ≥ 2.5% were 51.3% vs. 33.9% [difference from metformin 17.4% (9.6%, 25.1%)]. Not counting patients who experienced one or more events of hypoglycaemia (3.4% in the saxagliptin plus metformin group vs. 4.0% in the placebo plus metformin group), the rates of response defined by HbA_1c_ reduction ≥ 2.0% were 65.7% with saxagliptin plus metformin vs. 47.3% with metformin monotherapy [difference from metformin 18.4% (10.6%, 26.0%)]. Excluding hypoglycaemia, rates of response defined by HbA_1c_ reduction ≥ 2.5% were 49.3% vs. 31.6% [difference from metformin 17.7% (10.0%, 25.2%)].

**Table:** Glycaemic responses at 24 weeks with saxagliptin + metformin* as initial combination therapy vs. metformin* monotherapy in drug-naive patients

		Response rates among all assessed patients		Response rates in patients who did not experience hypoglycaemia	
Criterion for response	Treatment groups	*n*/*N* (%)	Difference (95% CI) vs. PBO + MET	*n*/*N* (%)	Difference (95% CI) vs. PBO + MET
HbA_1c_ reduction from BL ≥ 2.5%	MET (*n *= 328)	106/313 (33.9)	–	99/313 (31.6)	–
	SAXA 5 + MET (*n *= 320)	157/306 (51.3)	17.4% (9.6%, 25.1%)	151/306 (49.3)	17.7% (10.0%, 25.2%)
HbA_1c_ reduction from BL ≥ 2.0%	MET (*n *= 328)	156/313 (49.8)	–	148/313 (47.3)	–
	SAXA 5 + MET (*n *= 320)	209/306 (68.3)	18.5% (10.7%, 26.0%)	201/306 (65.7)	18.4% (10.6%, 26.0%)
HbA_1c_ reduction from BL ≥ 1.5%	MET (*n *= 328)	200/313 (63.9)	–	190/313 (60.7)	–
	SAXA 5 + MET (*n *= 320)	233/306 (76.1)	12.2% (5.0%, 19.4%)	223/306 (72.9)	12.2% (4.7%, 19.5%)
HbA_1c_ reduction from BL ≥ 1.0%	MET (*n *= 328)	243/313 (77.6)	–	231/313 (73.8)	–
	SAXA 5 + MET (*n *= 320)	268/306 (87.6)	9.9% (4.0%, 15.9%)	257/306 (84.0)	10.2% (3.8%, 16.6%)
HbA_1c_ reduction from BL ≥ 0.7%	MET (*n *= 328)	256/313 (81.8)	–	ND	
	SAXA 5 + MET (*n *= 320)	286/306 (93.5)	17.8% (12.3%, 23.4%)		
HbA_1c_ reduction from BL ≥ 0.5%	MET (*n *= 328)	270/313 (86.3)	–	ND	
	SAXA 5 + MET (*n *= 320)	293/306 (95.8)	15.3% (10.5%, 20.5%)		

BL, baseline; CI, confidence interval; HbA_1c_, glycated haemoglobin; MET, metformin; ND, not determined; PBO, placebo; SAXA 5, saxagliptin 5 mg once daily. *n*/*N*, number of responders/number assessed.

* Metformin titrated up to target dosage of 2000 mg/day.

### Correlation between baseline characteristics and glycaemic response

Assessment of response rates (HbA_1c_ reduction ≥ 1% in the add-on studies and ≥ 2% in the initial combination study) in patients stratified by baseline demographic and clinical categorical covariates (sex, age, duration of diabetes, BMI, HbA_1c_, C-peptide AUC, insulin AUC, glucagon AUC, glucose AUC and insulin secretion) suggested only isolated correlations with response rates. The study of add-on to metformin suggested a correlation for saxagliptin 2.5 mg vs. placebo only in baseline glucose AUC (odds ratio, 1.07; p < 0.05). The study of add-on to glyburide suggested a correlation for saxagliptin 2.5 mg vs. uptitration of glyburide only among patients with BMI ≥ 30 kg/m^2^ vs. BMI < 30 kg/m^2^ (odds ratio, 1.39; p < 0.05). The study of add-on to a TZD showed no correlations between baseline covariates and treatment response rates. The study of saxagliptin 5 mg plus metformin as initial combination therapy in drug-naive patients suggested a correlation for saxagliptin plus metformin vs. metformin monotherapy only for baseline HbA_1c_ (odds ratio, 3.08; p < 0.05). All other correlations between baseline covariates and treatment response in the four studies were not significant. With no consistent associations across doses or across studies with different background medications, it is likely that the cited isolated examples represent chance findings.

## Discussion

In a prospective observational study of the UKPDS participants 4, the incidence rates for any diabetes pathology-related end-point steadily increased with every 1% increase in HbA_1c_ level, such that there was a threefold increase in risk for diabetes complications (myocardial infarction, sudden death, angina, stroke, renal failure, lower extremity amputation or death from peripheral vascular disease, death from hyperglycaemia or hypoglycaemia, heart failure, vitreous haemorrhage, retinal photocoagulation and cataract extraction) over the HbA_1c_ range of median values of 5.6–10.6%. Importantly, there was no HbA_1c_ threshold at which the risk trajectory changed. Thus, meaningful reductions in HbA_1c_ can be clinically beneficial even if the goal of maintaining HbA_1c_ below 7% is not achieved.

The purpose of this study was to assess the ability of treatment with saxagliptin to provide reductions in HbA_1c_ ≥ 1% [identified as clinically consequential in the UKPDS 4] and other large mean reductions in HbA_1c_ in patients with T2DM, while maintaining a low incidence of hypoglycaemia. The original published reports of these studies demonstrated that mean reductions in HbA_1c_ were significantly greater with saxagliptin than with comparators. Likewise, these *post hoc* analyses show significantly higher rates of large glycaemic response with saxagliptin 2.5 or 5 mg once daily vs. placebo as an add-on to another antihyperglycaemic agent (large response defined as HbA_1c_ reduction ≥ 1%), and with saxagliptin 5 mg once daily plus metformin as initial combination therapy vs. metformin monotherapy in drug-naive patients (defined as HbA_1c_ reduction ≥ 2%).

Although DPP-4 inhibitors have been associated with mean reductions of 0.5–0.8% in HbA_1c_ 27, in the present analysis, HbA_1c_ reductions ≥ 1% were achieved by 18–29% more patients with T2DM receiving saxagliptin 2.5 or 5 mg as an add-on to another antihyperglycaemic agent than placebo. Moreover, reductions ≥ 2% were achieved by 18% more drug-naive patients receiving initial combination therapy with saxagliptin 5 mg and metformin than metformin monotherapy. Among patients receiving saxagliptin and achieving these reductions without experiencing an event of hypoglycaemia, response rates were 13–29% higher than placebo in the add-on studies and 18% higher than metformin alone in the initial combination with metformin study.

A further objective of this analysis was to understand predictors of larger than mean glycaemic response based on baseline patient demographics, which could assist in identifying patients in whom a robust response could be expected. Unfortunately, no consistent associations across doses or across studies with different background medications were found among the principal demographic and clinical covariates employed in these studies. Given the dichotomous end-point and a relatively limited sample size for detecting interactions, the ability to detect differential treatment effects by subgroup is limited in this dataset. Larger patient populations, such as those for DPP-4 outcome studies such as SAVOR 28, may provide more power to identify subpopulations that may experience more glycaemic benefit.

Patients for whom metformin is contraindicated, such as those with renal disease or renal dysfunction because of the risk of lactic acidosis, or for whom metformin should not be initiated, such as patients ≥ 80 years of age unless adequate renal function has been demonstrated, are candidates for treatment with DPP-4 inhibitors 29. Limited conclusions can be made about saxagliptin efficacy and tolerability in these patient populations from the current analyses because in the included studies, less than 20% of patients were 65 years or older, and patients with abnormal renal function were excluded. However, in a 12-week study 30 with 40-week extension 31 in patients with T2DM and moderate to severe renal impairment or end-stage renal disease, saxagliptin was effective in reducing HbA_1c_ vs. placebo (placebo-corrected, adjusted mean change from baseline −0.42% at 12 weeks and –0.73% at 52 weeks) and was generally well tolerated. Sitagliptin and linagliptin have also provided effective glycaemic control with good overall tolerability in patients with T2DM and moderate or severe renal insufficiency, with mean placebo-corrected reductions from baseline of 0.4–0.6% at 12 weeks, which were sustained up to 1 year 32–33. DPP-4 inhibitors are also effective in reducing HbA_1c_ as monotherapy or an add-on therapy in elderly patients, with reported reductions ranging from approximately 0.5 to 1.2% 34–38. In addition, results of a meta-analysis of trials suggest that older age is a predictor of greater efficacy of DPP-4 inhibitors in T2DM 39.

Several classes of antihyperglycaemic agents can produce meaningful reductions in HbA_1c_, but they differ in terms of associated risk, including risk of hypoglycaemia. Sulphonylureas have a high risk for hypoglycaemia whereas DPP-4 inhibitors have a low risk 40. The combination of saxagliptin with a sulphonylurea was associated with increased reports of hypoglycaemia 15, compared with that observed with saxagliptin plus metformin combination 16. Therefore, it is not surprising that in the present *post hoc* analyses, the differences between overall rates of glycaemic response and rates of response without experiencing hypoglycaemia were greater in the study of saxagliptin add-on to glyburide than in the other studies (Tables 4), reflecting the higher incidence of hypoglycaemia associated with the sulphonylurea. It is therefore recommended that the dose of sulphonylurea be lowered when used concomitantly with saxagliptin or other DPP-4 inhibitors 26. The *post hoc* analyses presented here indicate that saxagliptin used as an add-on to another non-insulin antihyperglycaemic agent (except perhaps a sulphonylurea) can achieve large decreases in HbA_1c_ with minimal risk of hypoglycaemia. This outcome may be attributable to its glucose-dependent mechanism.

Limitations of the analyses presented include the fact that they were performed *post hoc*, using last observations carried forward. The criteria used in this analysis to define a large reduction in HbA_1c_ were based on general clinical expectations of what constitutes a large glycaemic response and changes in HbA_1c_ that have been documented to reduce T2DM-related complications 4. In addition, decreases in HbA_1c_ should always be considered in the context of baseline values in the studies; therefore, the reported outcomes may not be applicable to patients with baseline HbA_1c_ outside the range required for study inclusion. In the saxagliptin studies, the ADA guidelines on diet and exercise were supported. It is likely (based on reductions in HbA_1c_ with placebo) that diet and exercise compliance improved between the randomisation and primary analysis point. Finally, the analyses offer no insight into durability of the glycaemic effect assessed here.

## Conclusions

Reduction in HbA_1c_ can reduce the incidence or severity of complications related to T2DM, even if the goal of maintaining HbA_1c_ < 7% is not achieved. The present *post hoc* analyses of studies of saxagliptin as an add-on therapy to another antihyperglycaemic drug and as initial combination therapy with metformin in drug-naive patients assessed the proportions of T2DM patients who achieved glycaemic responses larger than the mean reductions expected with a DPP-4 inhibitor (≥ 1.0% in the add-on studies; ≥ 1.0% to ≥ 2.5% in the initial combination study). The analyses show that the incidence of hypoglycaemia is low with saxagliptin and that the proportions of patients who achieve large glycaemic responses are significantly higher with saxagliptin than with the comparator regimens, even when patients who experience hypoglycaemia are not counted as responders.

## Author contributions

All authors of this manuscript had full access to the data and participated in the concept, design and drafting of this manuscript.
